# Heterogeneous diffusion in aerobic granular sludge

**DOI:** 10.1002/bit.27522

**Published:** 2020-08-06

**Authors:** Lenno van den Berg, Catherine M. Kirkland, Joseph D. Seymour, Sarah L. Codd, Mark C. M. van Loosdrecht, Merle K. de Kreuk

**Affiliations:** ^1^ Department of Water Management Delft University of Technology Delft The Netherlands; ^2^ Center for Biofilm Engineering Montana State University Bozeman Montana; ^3^ Department of Civil Engineering Montana State University Bozeman Montana; ^4^ Department of Chemical and Biological Engineering Montana State University Bozeman Montana; ^5^ Department of Mechanical and Industrial Engineering Montana State University Bozeman Montana; ^6^ Department of Biotechnology Delft University of Technology Delft The Netherlands

**Keywords:** aerobic granular sludge, diffusion, granule structure, heterogeneity, NMR

## Abstract

Aerobic granular sludge (AGS) technology allows simultaneous nitrogen, phosphorus, and carbon removal in compact wastewater treatment processes. To operate, design, and model AGS reactors, it is essential to properly understand the diffusive transport within the granules. In this study, diffusive mass transfer within full‐scale and lab‐scale AGS was characterized with nuclear magnetic resonance (NMR) methods. Self‐diffusion coefficients of water inside the granules were determined with pulsed‐field gradient NMR, while the granule structure was visualized with NMR imaging. A reaction‐diffusion granule‐scale model was set up to evaluate the impact of heterogeneous diffusion on granule performance. The self‐diffusion coefficient of water in AGS was ∼70% of the self‐diffusion coefficient of free water. There was no significant difference between self‐diffusion in AGS from full‐scale treatment plants and from lab‐scale reactors. The results of the model showed that diffusional heterogeneity did not lead to a major change of flux into the granule (<1%). This study shows that differences between granular sludges and heterogeneity within granules have little impact on the kinetic properties of AGS. Thus, a relatively simple approach is sufficient to describe mass transport by diffusion into the granules.

## Graphical abstract

Aerobic granules are heterogeneous structures, but the impact of heterogeneity on diffusive mass transport is not well understood. Van den Berg and coworkers used a granule‐scale reaction‐diffusion model to test the impact of various modes of heterogeneous diffusion on granule performance. Surprisingly, the impact of heterogeneous diffusion was only minor (< 5%).

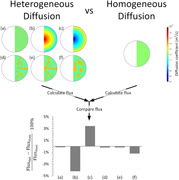

## 1 INTRODUCTION

Aerobic granular sludge (AGS) is an advanced technology for the treatment of wastewater. Aerobic granules are compact microbial aggregates, which allow simultaneous removal of chemical oxygen demand (COD), nitrogen, and phosphate, and have excellent settling properties (Adav, Lee, Show, & Tay, 2008; Kishida, Tsuneda, Kim, & Sudo, 2009; de Kreuk, Heijnen, & van Loosdrecht, 2005). AGS technology has a reduced footprint and energy requirement compared to conventional activated sludge processes (de Bruin, de Kreuk, van der Roest, Uijterlinde, & van Loosdrecht, 2004). Since the first full‐scale application of AGS in 2005, many AGS installations have been built (Pronk, Giesen, Thomphson, Robertson, & van Loosdrecht, 2017; van der Roest, de Bruin, Gademan, & Coelho, 2011).

To treat the wastewater in AGS installations, contaminants have to be transferred from the bulk liquid to the microorganisms that are located within the granules. Due to the compact and dense nature of the granules (Nor‐Anuar, Ujang, van Loosdrecht, de Kreuk, & Olsson, 2012), diffusion is generally the predominant mass transfer mechanism. Diffusion of different solutes in granules can be relatively slow compared to the volumetric reaction rates. A variety of chemical gradients within the granule (e.g., substrate concentration, oxygen concentration, and pH) arise as a result. These gradients directly impact the conversion processes in the granular sludge reactors. The simultaneous nitrification and denitrification require a careful balance between the aerobic and the anoxic volume of the granules (Di Bella & Torregrossa, 2013; Mosquera‐Corral, de Kreuk, Heijnen, & van Loosdrecht, 2005; Yilmaz, Lemaire, Keller, & Yuan, 2008). These processes can only occur at the same time within a granule if the nitrification reaction is diffusion limited (Daigger & Littleton, 2014). On the other hand, mass transfer limitation can lead to filamentous outgrowth or hollow cores in the granule. Both phenomena have been shown to notably reduce reactor performance (de Kreuk, Kishida, Tsuneda, & van Loosdrecht, 2010; Zheng, Yu, Liu, & Liu, 2006). Therefore, understanding of the diffusive transport within granules is essential to operate, design, and model AGS reactors.

Diffusion has been extensively studied in biofilm systems, with methods such as the diffusion cell (Horn & Morgenroth, 2006; Pu & Yang, 1988) and microelectrodes (Chiu et al., 2006; Chiu, Chen, Lee, Wang, & Lai, 2007a). However, neither of these methods can detect heterogeneities of diffusion coefficients from the surface to the inner parts of a biofilm. Pulsed‐field gradient nuclear magnetic resonance (PFG‐NMR) is an alternative method that overcomes these disadvantages. It can determine the displacement of hydrogen‐bearing molecules in a sample. This displacement can be related to the self‐diffusion of water molecules, since water is the most abundant hydrogen‐bearing molecule in biofilms. Furthermore, with PFG‐NMR one can distinguish water molecules based on their local physical and chemical environment in a biofilm. The self‐diffusion coefficient of water in biofilms has been found to be a good indicator of the diffusion coefficient of glucose (Beuling et al., 1998) and oxygen (Wieland et al., 2001). Previous work with PFG‐NMR has focused on artificial and natural biofilms (Beuling et al., 1998; Hornemann et al., 2008; Renslow et al., 2010) and various types of anaerobic granular sludge (Gonzalez‐Gil et al., 2001; Lens et al., 2003; Lens, Pol, Lettinga, & Van As, 1997). It was observed that both biofilms and anaerobic granules were not homogeneous and contained a distribution of diffusion coefficients. Lens et al. (2003) reported that the type of wastewater and operational conditions influenced the diffusional properties of the granular sludge. However, it is unclear to what extent diffusivity in biofilms and anaerobic granules is similar to that in aerobic granules.

The aim of this study was to characterize the diffusional properties of AGS. We used PFG‐NMR to measure effective diffusion coefficients in granular sludges from full‐scale and lab‐scale AGS reactors. Furthermore, we investigated the presence of heterogeneous diffusion within the granules and its impact on process engineering of granular sludge processes. Lastly, we provided a recommendation on how to include diffusion in the analysis of AGS kinetic properties and in AGS modeling.

## 2 MATERIALS AND METHODS

### 2.1 Source of biomass

AGS was collected from full‐scale AGS plants in Garmerwolde, Vroomshoop, and Simpelveld, all located in the Netherlands. The plants were designed by Royal HaskoningDHV under the trade name Nereda®. The plants treat domestic wastewater with influent concentrations as shown in Table 1. They are operated with biological phosphate removal and an average solids retention time of 20–50 days. Laboratory‐scale AGS was taken from a fresh‐water reactor and a sea‐water reactor, both fed with acetate as sole carbon source. Reactor operation is described elsewhere (de Graaff, van Dijk, van Loosdrecht, & Pronk, 2018). All samples were rinsed with tap water to remove the majority of flocculent biomass. The granules were stored in tap water at 4°C for up to 2 months. No changes in self‐diffusion coefficients were observed during this period. For the NMR measurements, the granules were poured onto a petri‐dish and granules with a size of roughly 1–3 mm in diameter were manually selected.

**Table 1:** Average sludge loading and influent concentrations of the reactors from which the aerobic granular sludge was harvested

	Sludge loading (kg/kgTSS/d)		Influent (mg/L)			
	COD	BOD_5_	COD	BOD_5_	SS	Reference
Laboratory‐scale reactors						
Fresh water	0.18	0.18	366	366	–	de Graaff et al. (2018)
Saline water	0.18	0.18	366	366	–	de Graaff et al. (2018)
Full‐scale reactors						
Nereda Garmerwolde	0.10	0.04	506	224	236	Pronk et al. (2015)
Nereda Vroomshoop	0.09	0.03	720	263	317	Pronk et al. (2017)
Nereda Simpelveld	0.04	0.01	300	124	169	

Abbreviations: BOD_5_, biochemical oxygen demand in 5 days; COD, chemical oxygen demand; SS, suspended solids; TSS, total suspended solids.

### 2.2 NMR measurements of diffusion coefficients

Self‐diffusion coefficients of water molecules within granules and within bulk water were measured using the pulsed‐field gradient stimulated echo (PFG‐STE) sequence (Stejskal & Tanner, 1965). The PFG‐STE measurements were carried out at room temperature (20 ± °C) with a 250‐MHz superconducting magnet (Bruker Avance III, Bruker BioSpin GmbH, Rheinstetten, Germany). The magnet was equipped with a Diff30 probe (17 T/m maximum gradient). For the diffusion measurement, granules were stacked in a 5‐mm NMR tube without excess water to maximize the signal obtained from the granules relative to bulk water signal. Roughly, 20–30 granules were within the sensitive region of the NMR spectrometer, and thus contributed to the NMR signal. Typical acquisition parameters were as follows: 5,000 Hz spectral width, 1 ms gradient pulse duration (δ), 40 ms gradient pulse separation (diffusion time Δ), 1 s repetition time, 0–2.65 T/m diffusion gradient amplitude (*g*, linear in 128 steps), and 32 averages.

Fitting of the normalized NMR signal $S/S_{0}$ with a monoexponential or biexponential model will yield the diffusion coefficient(s) of the diffusing population(s) within the sample. Here, a biexponential model was used to fit the signal of the granular sludge samples:

$$\frac{S}{S_{0}}=A_{1}\text{exp}\left(-\gamma^{2}g^{2}\delta^{2}D_{1}\left(\Delta -\frac{\delta }{3}\right)\right)+A_{2}\text{exp}\left(-\gamma^{2}g^{2}\delta^{2}D_{2}\left(\Delta -\frac{\delta }{3}\right)\right),$$

where *A*_1_ and *A*_2_ correspond to the relative size of two diffusing populations, *D*_1_ and *D*_2_ their respective diffusion coefficient (m^2^/s), *γ* the gyromagnetic ratio (MHz/T), *g* the gradient amplitude (T/m), δ the gradient pulse duration (ms), and Δ the diffusion time (ms). A plot of a typical normalized NMR signal with monoexponential and biexponential fits is shown in Figure S1.

The normalized NMR signal was also processed with the 1D Inverse Laplace Transform, which uses a nonnegative least squares fitting function with a regularization parameter to minimize the error in the solution (Callaghan, Godefroy, & Ryland, 2003). Here, the data were transformed with a regularization parameter α of 1 × 10^8^and 64 steps. The regularization parameter was chosen based on the approach of Provencher (1982). This process yields a one‐dimensional (1D) diffusion coefficient distribution. However, due to the ill‐posed nature of the Inverse Laplace Transform, the distribution is only an approximation and should be interpreted accordingly.

Self‐diffusion coefficients of fresh and saline water without granules present were measured with the same acquisition parameters, but a monoexponential model was used for data analysis. These self‐diffusion coefficients of water without granules were used to quantify the impact of the granule matrix on the mobility of water molecules.

### 2.3 MRI measurements of granule structure

The granule structure was characterized with magnetic resonance imaging (MRI) experiments. The goal of this experiment was to obtain transverse relaxation times (*T_*2*_*) at different locations throughout the granule. The *T*_*2*_ time is a measure of how fast the NMR signal loses phase coherence after an excitation pulse. The relaxation time depends on the local physical‐chemical environment in which different water populations exist (Callaghan, 1993). Since water molecules in a granule are generally less mobile, they will have a shorter *T*_*2*_ time than water molecules in bulk liquid (Hoskins, Fevang, Majors, Sharma, & Georgiou, 1999). Different locations in the granule can have different local environments (e.g., cell density, extracellular polymeric substances [EPS] density, paramagnetic ions) and therefore different *T*_*2*_ values. Visualizing these differences in a *T*_*2*_ map can be used to characterize the granule structure (Kirkland et al., in press; Seymour, Codd, Gjersing, & Stewart, 2004).

The MRI experiments were performed with a 250‐MHz superconducting magnet (Bruker Avance III, Bruker BioSpin GmbH, Rheinstetten, Germany). The magnet was equipped with a high‐power probe, micro5 gradient set (2.81 T/m maximum gradient), and a 5‐mm radio‐frequency coil. A stack of 5–10 granules was placed in a 5‐mm NMR tube filled with tap water. A multislice multiecho (MSME) imaging sequence was used to acquire *T*_*2*_‐weighted images. Typical acquisition parameters were as follows: 5 s repetition time, 5.04 ms echo time, 8 echoes, 16 averages, 50 kHz spectral width, 39 × 39 × 200 μm resolution, and 5 × 5 mm field‐of‐view. The MSME produces a stack of 2D images showing the NMR signal amplitude per pixel at each image acquisition time. Because the NMR signal amplitude decays with time, fitting the signal attenuation in each pixel ultimately yields the effective *T*_*2*_ relaxation time in each pixel (Edzes, van Dusschoten, & Van As, 1998). Here, 2D maps of the *T*_*2*_ relaxation times were obtained with Prospa v3.13 (Magritek).

### 2.4 NMR measurements to correlate diffusion with structure

A 2D correlation experiment was conducted to relate diffusion to *T*_*2*_ relaxation. During the experiment, each water molecule in the granular sludge sample will diffuse at a certain rate. Simultaneously, each water molecule will experience a *T*_*2*_ relaxation that is indicative of its local environment. With a correlation experiment, the diffusion and relaxation rate are measured for each water molecule. It should be noted that, unlike in the MRI experiment, the *T*_*2*_ values are not spatially resolved. Correlation of the diffusion and *T*_*2*_ relaxation rate can give valuable insight into the relationship between structure (by *T*_*2*_ relaxation) and diffusion in the granular sludge sample. The correlation experiment was carried out with a PFG‐STE sequence, followed by a Carr–Purcell–Meiboom–Gill sequence (Callaghan, 2011). The data were processed in Matlab with the 2D Inverse Laplace Transform, which is similar to the aforementioned 1D Inverse Laplace Transform (Callaghan et al., 2003). Here, the data were transformed with a regularization parameter α of 1 × 10^8^and 64 steps. The regularization parameter was chosen based on the approach of Provencher (1982).

### 2.5 Granule‐scale reaction‐diffusion model

**Figure 1:**
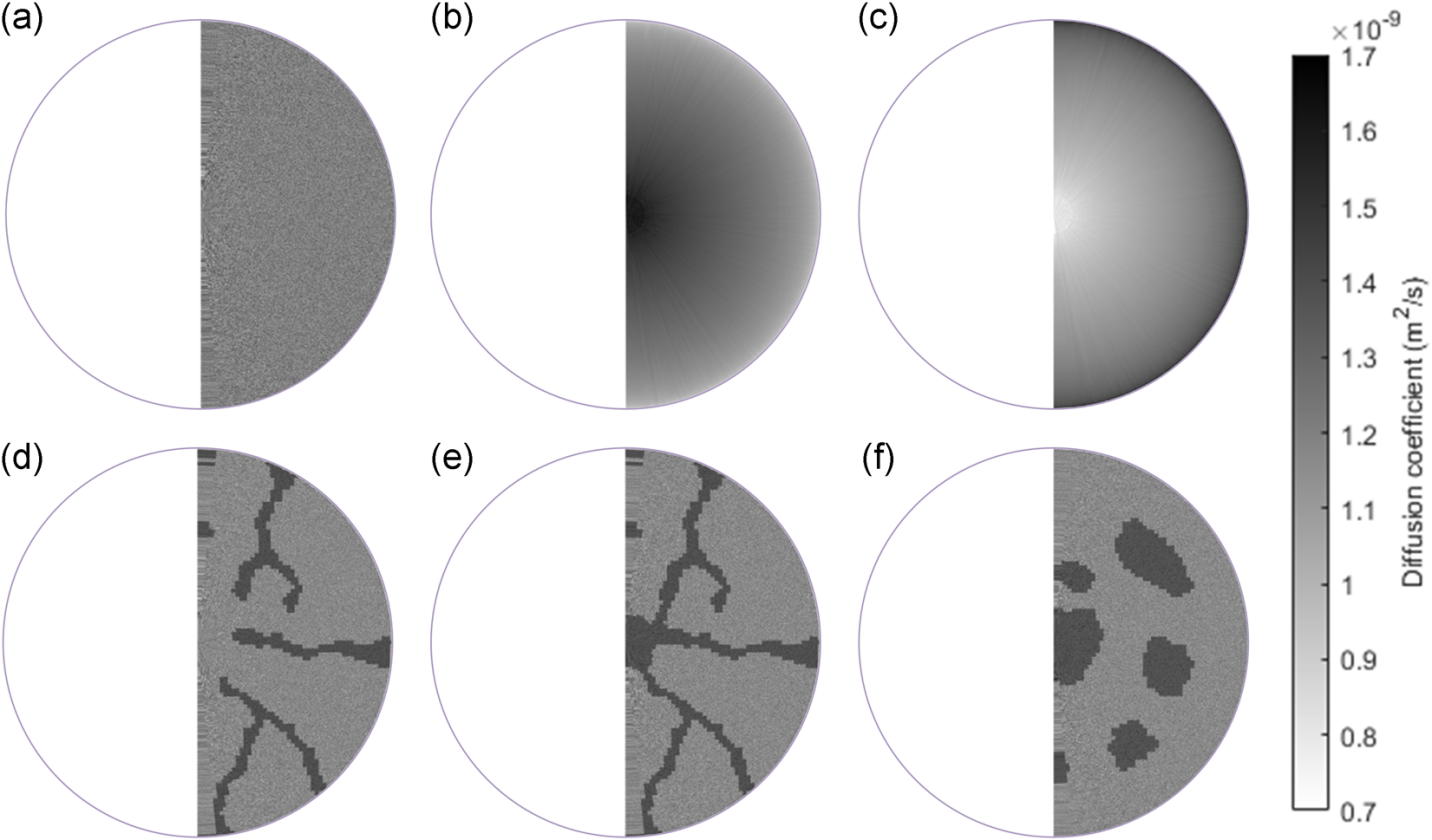
Spatial distribution of diffusion coefficients of oxygen for six different scenarios (all with 600,000 grid points). The color bar indicates the diffusion coefficient of oxygen. An axisymmetric granule model was used for simplicity, meaning that the granule was assumed to be rotationally symmetric around the vertical axis through the granule center. (a) Randomly distributed diffusion coefficient. (b) Concentric diffusion coefficient profile with decreasing diffusion coefficient toward the granule surface. (c) concentric diffusion coefficient profile with increasing diffusion coefficient toward the granule surface. (d) Channels with increased diffusion coefficient. (e) Channels and core with increased diffusion coefficient. (f) Voids throughout the granule with increased diffusion coefficient

A 2D axisymmetric, steady‐state, reaction‐diffusion granule model was set up in COMSOL Multiphysics. Heterotrophic oxidation of organic matter was used as model reaction, with Monod kinetics and oxygen as single limiting substrate. Model parameters were derived from the first biofilm benchmark problem (Morgenroth et al., 2004) and are as follows: *q*_max_ = 3.54 gO_2_·gCOD·d, *C*_X_ = 10,000 gCOD/m^3^, *K* = 0.2 gO_2_/m^3^. The granule radius was set to 0.55 mm, with a bulk oxygen concentration of 2 g/m^3^. Six different scenarios were created with respect to the heterogeneous distribution of the diffusion coefficient (see Figure 1). The flux of oxygen into the granule for each case was calculated in Comsol with an integration of the diffusive flux over the granule surface. The flux deviation was calculated as the relative difference between the flux for each case and flux obtained in a scenario with a homogeneous diffusion coefficient. In all scenarios, the diffusion coefficient was normally distributed, with a mean of 1.4 × 10^−9^ m^2^/s (based on the result of this study, see Table 2) and a relative standard deviation of 10%. Convective mass transfer was not included. The granules were discretized with 600,000 grid points based on equal volumes. A sensitivity analysis was carried out with discretization with 60 and 6,000 grid points.

**Table 2:** Overview of the water self‐diffusion coefficient of the granular sludges

	*D*	*D*/*D_aq_*
	10^−9^ m^2^/s	–
Lab‐scale reactors		
Fresh water	1.44 ± 0.01	0.71
Saline water	1.32 ± 0.03	0.69
Full‐scale reactors		
Nereda Garmerwolde	1.39 ± 0.04	0.69
Nereda Vroomshoop	1.33 ± 0.05	0.66
Nereda Simpelveld	1.32 ± 0.10	0.65

*Note:* The relative diffusivity (*D/D*_*aq*_) was based on the self‐diffusion coefficient of fresh water (*D*_*aq*_). For the granules grown in saline water, the self‐diffusion coefficient of saline water was used instead. NMR measurements revealed the self‐diffusion coefficient of fresh‐water to be 2.03 × 10^−9^ m^2^/s and of saline water to be 1.91 × 10^−9^ m^2^/s. Diffusivity values are given as mean ± standard deviations from triplicate experiments.

## 3 RESULTS

### 3.1 Self‐diffusion of water in granular sludge

The self‐diffusion coefficients of the granular sludges were analyzed with a biexponential model. For all sludges, the NMR signal originated from a large amount of mobile water (∼95% of the signal) and a smaller amount of less mobile water (roughly 5% of the signal). The mobile water had an average diffusion coefficient between 1.3 × 10^−9^ and 1.5 × 10^−9^ m^2^/s. The diffusivity of the less mobile water was around 1.0 × 10^−10^ m^2^/s. The diffusion coefficient distributions determined with the Inverse Laplace Transform can be found in Figures S1 and S2.

The diffusional properties of the granular sludges were determined in triplicate (i.e., three NMR tubes with 20–30 granules each). The average diffusion coefficients can be found in Table 2. The standard deviation within the triplicates was small and exceeded 5% only for the Simpelveld granules. The diffusional properties of the granular sludges did not depend on their origin, based on a one‐way analysis of variance (*F*(4,10) = 2.57, *p* = .10).

### 3.2 Granule structure

**Figure 2:**
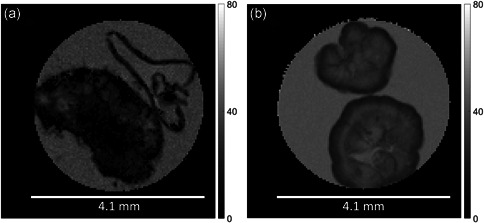
Typical effective *T*_*2*_ map for a Garmerwolde full‐scale granule (a) and a saline lab‐scale (b) granule. The scale bar indicates effective *T*_*2*_ in ms. The spatial resolution is 39 × 39 × 100 µm. Lighter regions correspond to a higher *T*_*2*_ (more water‐like), while darker regions correspond to a lower *T*_*2*_ (more solid‐like)

A *T*_*2*_ map reveals the granule structure, by showing the local *T*_*2*_ values throughout the granule. Roughly, six granules were imaged per sludge source. Overall structural features were constant between different granules of the same origin (data not shown), similar to what was found by Kirkland et al. In Figure 2, only two *T*_*2*_ maps are presented: one typical of full‐scale granules and one typical of lab‐scale granules. Maps for other granules can be found in Figures S3–S7.

The *T*_*2*_ map of a full‐scale granule shows that there is a range of *T*_*2*_ values within a single granule, confirming that water is present in different environments (see Figure 2a). The full‐scale granule has a heterogeneous structure, with regions that have a *T*_*2*_ close to that of bulk water, and with regions in which water is either not present or strongly restricted in mobility. No apparent ultrastructure could be observed (e.g., concentric, cluster‐like structure). The surface of the granule is not smooth, possibly due to the presence of protozoa clusters or filamentous outgrowths (Pronk et al., 2015). The *T*_*2*_ map of lab‐scale granules reveals much less heterogeneity (see Figure 2b). The variation in *T*_*2*_ values in the granules showed a concentric pattern. The core of the granules has a *T*_*2*_ close to that of the bulk water, which suggests that the granules are filled with water and effectively hollow.

**Figure 3:**
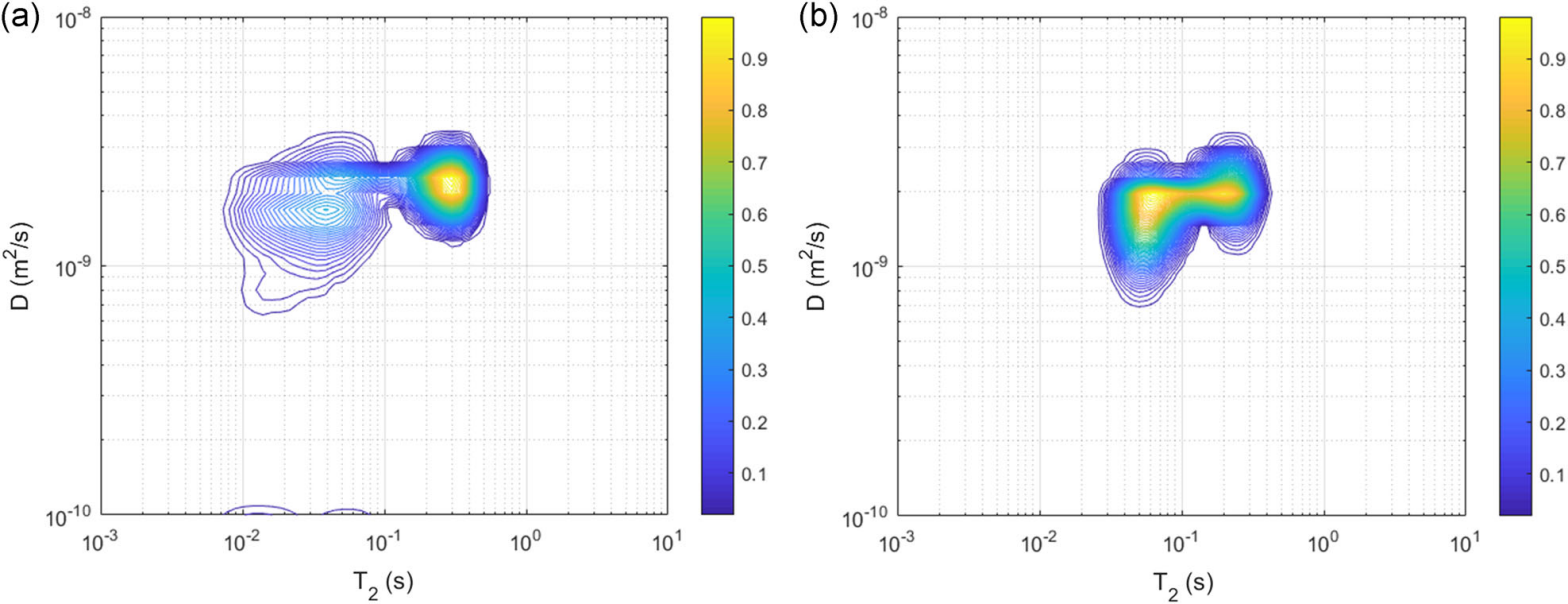
Typical D‐*T*_*2*_ correlation for Garmerwolde full‐scale granular sludge (a) and saline lab‐scale (b) granular sludge. The color bar represent signal intensity in arbitrary units [Color figure can be viewed at wileyonlinelibrary.com]

The heterogeneity of the *T*_*2*_ maps can be related to diffusional properties with a *D*‐*T*_*2*_ correlation experiment. This experiment correlates the *T*_*2*_ of water molecules to the diffusion coefficient of those molecules. It includes all the observable water molecules in a granular sludge sample. In Figure 3, typical *D*‐*T*_*2*_ correlations are shown for full‐scale and lab‐scale granular sludge. It should be noted that the *T*_*2*_ values in the D‐*T*_*2*_ correlation experiment are almost an order of magnitude smaller than the *T*_*2*_ values in the *T*_*2*_ maps (cf., Figures 2 and 3). This is not unexpected, since the *T*_*2*_ maps represent effective *T*_*2*_, while the *D*‐*T*_*2*_ correlation represents true *T*_*2*_. The effective *T*_*2*_ is reduced due to the imaging gradients required for the *T*_*2*_ maps (Edzes et al., 1998). Therefore, direct comparison of the different *T*_2_ values is not possible.

A range of *T*_*2*_ values was present in the granular sludge, again revealing the presence of a range of physical and chemical environments. The *T*_*2*_ values of the full‐scale granular sludge were lower than those of lab‐scale granular sludge, indicating that water was more restricted in the full‐scale sludge. Note that neither free water (the NMR tube did not contain excess water during these measurements) nor intracellular water (which has a relatively small signal) contributed to the correlation. The correlation also showed a range of diffusivity values, although with a narrower distribution. Clearly, water within granular sludge did not have a single, discrete diffusion coefficient, but rather a diffusion coefficient distribution. However, there was no clear correlation between *T*_*2*_ and diffusivity.

### 3.3 Granule heterogeneity model

**Figure 4:**
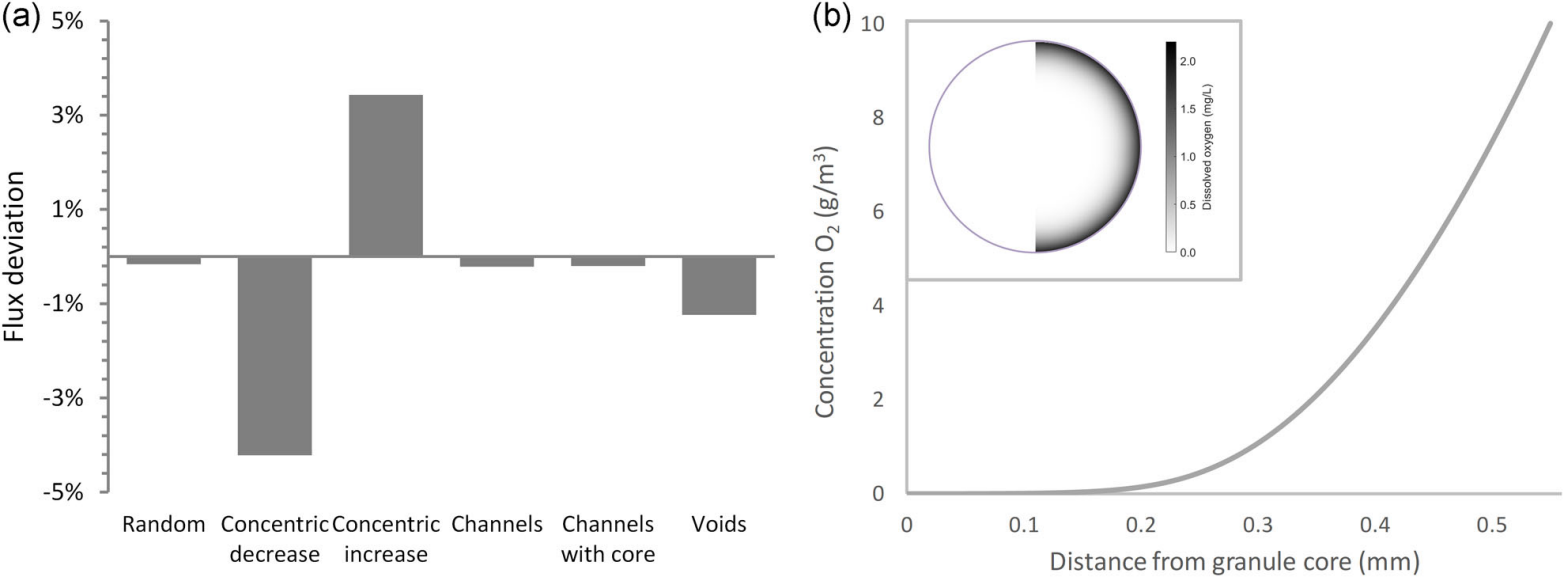
Deviation of steady‐state flux of solute into the granule for the six different heterogeneous diffusion scenarios compared to homogeneous diffusion as defined in Figure 1. (a) Typical 1D steady‐state concentration profile in a scenario with homogeneous diffusion, showing the limited penetration in these simulations, as well as a 2D steady‐state concentration profile for the channels scenario (b)

The impact of different models of heterogeneous diffusion on the flux of a model substrate into a granule was evaluated with a reaction‐diffusion model. In all six diffusion models, oxygen penetrated only the outer part of the granule, as can be seen in the 1D oxygen profile of Figure 4b. The full spatial oxygen profiles for each diffusion model showed no discernible difference and therefore only the spatial profile for the channels scenario is shown (insert of Figure 4b). For the six heterogeneous diffusion models shown in Figure 1, the deviations in flux (compared to the flux for homogeneous diffusion) was always smaller than 5% (see Figure 4a). Channels or voids with a higher diffusivity apparently only slightly enhance diffusive mass transfer into the granule. When the diffusion coefficient varied with the granule radius (cases b and c), the largest deviation in flux was observed. A low diffusivity near the granule surface resulted in a reduced flux (case b), while a higher diffusivity near the surface resulted in an increased flux (case c). In these simulations, the substrate only partially penetrated the granule, as can be seen in Figure 4b. To see the impact of penetration depth, we calculated the average diffusion coefficient of the penetrated volume (here defined as the volume where the local concentration was at least 1% of the bulk concentration) for the heterogeneous diffusion scenarios. There was a strong correlation (*R* ^2^ = .97) between the diffusion coefficient of the penetrated volume and the flux deviation (see Figure S8). It appears that the change in flux for heterogeneous diffusion is almost entirely caused by the change in average diffusion coefficient of the outer layer. The number of grid points only had a minor influence, as the differences between the simulations with 60, 6,000, and 600,000 grid points was maximally 0.2%.

## 4 DISCUSSION

### 4.1 Self‐diffusion coefficient of granular sludges

The PFG‐NMR experiments yielded two diffusing populations in each granular sludge. Previously, two similar populations have been reported as well in anaerobic granular sludges and a biofilm (Beuling et al., 1998; Hornemann et al., 2008; Lens et al., 2003). They identified the less mobile population as cell‐internal water. The *T*_*2*_ for the less mobile population that was obtained in our study, was similar to the intracellular water in anaerobic granular sludge (10–30 ms). A quantitative analysis of the intracellular volume is complex, due to the small signal size, short *T*_*2*_ value, and the permeability of the bacterial cell wall to water (Beuling et al., 1998). Therefore, in our study, the small population of less mobile protons is excluded from all analyses.

For the mobile population, self‐diffusivity of water in the granules is reduced compared to the self‐diffusivity of free water. The diffusion coefficients that were found in this study for AGS are in the same order of magnitude as the water self‐diffusion coefficients determined for different anaerobic and aerobic aggregates and biofilms (Beuling et al., 1998; Hornemann et al., 2008; Herrling et al., 2017; Lens et al., 2003, 1997; Phoenix & Holmes, 2008; Renslow, Babauta, Majors, & Beyenal, 2013; Renslow et al., 2010; Vogt, Flemming, & Veeman, 2000).

Lens et al. (2003) have shown with PFG‐NMR that operational conditions can influence the diffusional properties of anaerobic granular sludge. No such effect was found for the AGS that was analyzed in this study. The variation in operating conditions between the granular sludges from the full‐scale treatment plants was minimal, since all plants treat domestic wastewater with biological phosphate removal. However, the lab‐scale reactors were operated with notably different hydrodynamics, influent characteristics, and loading. The wet density of lab‐grown granules is typically reported to be around 1,040 kg/m^3^ (Etterer & Wilderer, 2001; Herrling et al., 2017; Winkler, Bassin, Kleerebezem, Van der Lans, & Van Loosdrecht, 2012), although the ash content plays a major role (Winkler, Kleerebezem, Strous, Chandran, & Van Loosdrecht, 2013). The wet density of granules from the Garmerwolde treatment plant was found to be around 1,020 kg/m^3^. Considering the observed differences in granule structure and density, it was expected that full‐scale and lab‐scale granular sludges would have different diffusive properties, but this was not the case.

The diffusivity in a granule depends on its structural properties. EPS and relatively impermeable cells within the granule hinder diffusing molecules. A molecule has to diffuse around EPS and cells, increasing the diffusive path length. The increase in path‐length is generally referred to as tortuosity. The impact of tortuosity on the effective diffusivity in a granule is given by the following relation (Epstein, 1989):

$$D_{\text{eff}}=\frac{1}{\tau^{2}}\times D_{\mathrm{aq}},$$

where $D_{\text{eff}}$ is the effective diffusivity in the granule, $D_{\mathrm{aq}}$ is the diffusivity in the bulk water phase, and *τ* is the tortuosity. The tortuosity is defined here as ratio of the actual path length over the Euclidian distance (shortest linear distance). If the effective diffusivity in a granule is known (e.g., measured with PFG‐NMR), the tortuosity of the granular sludge can be calculated. For the granular sludges used in this study, the tortuosity is roughly 1.2. This means that the actual path length that a water molecule has moved along is only 20% increased due to the presence of cells, inorganic material, and EPS. Since a granule consist mostly of water, it is not surprising that the tortuosity is relatively close to 1 (Etterer & Wilderer, 2001; Zheng & Yu, 2007).

The diffusion coefficient that is obtained with PFG‐NMR is the effective diffusivity (Beuling et al., 1998; Stewart, 1998). The effective diffusivity is generally used to describe transient penetration of a solute into a biofilm. However, for wastewater treatment applications, the (pseudo‐)steady‐state flux of some solute into a granule is generally more relevant. This flux is not described by the effective diffusivity, but rather by the effective diffusive permeability. Since the terminology can be confusing, we refer to Stewart (1998) for a detailed discussion of both parameters. Here, the emphasis is on the relation between both parameters:

$$D_{\text{eff}}=(1-\phi )\times D_{\text{eff}},$$

where *D*_eff_ is the effective diffusive permeability (Libicki, Salmon, & Robertson, 1988) and ϕ is the volume fraction occupied by cells and EPS. The effective diffusive permeability includes another effect of the granule matrix on the diffusivity: the volume exclusion by cells and EPS. Since a molecule cannot diffuse through the nonwater phase of a granule, the effective diffusive permeability is reduced.

To obtain effective diffusive permeability, further information on the porosity (1 − ϕ) of the granule is required. However, both the porosity and the tortuosity depend on the fraction of cells and EPS. Knowledge of one parameter can be used to estimate the other. For example, Zhang and Bishop (1994) found a tortuosity of 1.15 for biofilms with porosities of 0.84–0.93. Comparable values for porosities of AGS have been found by Etterer and Wilderer (2001) and Zheng and Yu (2007). Since the tortuosity of the granular sludges in this study was around 1.2, the porosities are most likely within this range as well. This indicates that the effective diffusive permeability is roughly 84–93% of the effective diffusivity.

### 4.2 Structural heterogeneity of granules

A range of *T*_*2*_ values was present and spatially distributed over the full‐scale granule, indicating the structural heterogeneity (Figure 2a). These findings are in line with the extensive study of granule structure by Kirkland et al. The *T*_*2*_ value measured using the imaging MSME sequence in a biofilm is influenced by the diffusion of the water molecules, presence of relaxation sinks, and magnetic field inhomogeneities (Brownstein & Tarr, 1979; Edzes et al., 1998; Godefroy, Korb, Fleury, & Bryant, 2001). Examples of relaxation sinks are bound water, paramagnetic impurities, and EPS. In a detailed study, Beuling et al. (1998) have shown the importance of the EPS content for the average relaxation time *T*_*2*_. An increase of the agar concentration from 1.5 to 4% wt/wt led to a decrease in relaxation time from 100 to 38 ms. The large impact of EPS on the relaxation time can be explained by the exchange of protons between water and EPS functional groups (e.g., −OH, −NH_2_, −SH groups). The impact of the EPS is a function of the amount of exchangeable protons, their local chemical environment (chemical shift), and their exchange rate (Hills, 1992). Thus, it can be deduced that the *T*_*2*_ relaxation time is not only impacted by the concentration of polymers, but also by the type of polymers. A different number of ionizable groups on a polymer will lead to a different *T*_*2*_ value.

Although many attempts have been made to relate the structure of AGS to mass transport within the granules (Chiu et al., 2007a; Chiu, Chen, Lee, Wang, & Lai, 2007b; Li, Liu, Shen, & Chen, 2008; Liu et al., 2010; Meyer, Saunders, Zeng, Keller, & Blackall, 2003; Tay, Ivanov, Pan, & Tay, 2002), the exact relationship remains unclear. These authors used (invasive) techniques that can either determine substrate diffusion into the granules (e.g., microelectrodes) or that can observe the heterogeneity inside the granules (e.g., confocal laser scanning microscopy methods). In this study, multiple attempts were made to obtain local diffusion coefficients in a granule with an apparent diffusion coefficient (ADC) map. An ADC map visualizes the heterogeneity of diffusion in a granule by displaying the ADC for each pixel in an image. However, due to rapid signal loss (short *T*_*2*_) in the granular sludge matrix, it was not possible to obtain useful ADC maps. As an alternative method to indicate the heterogeneity of diffusivity, *T*_*2*_ maps and *D*‐*T*_*2*_ correlations can be used. A *T*_*2*_ map shows local *T*_*2*_ values in a granule, while a *D*‐*T*_*2*_ correlation shows how diffusivity and *T*_*2*_ are related. A clear correlation implies that the heterogeneity for the diffusivity is similar to any heterogeneity visible in the *T*_*2*_ map. The *D*‐*T*_*2*_ correlation is much less impacted by the rapid signal loss since no imaging gradients are required in the experiment.

The *D*‐*T*_*2*_ correlations of both the full‐scale and lab‐scale granular sludge do not show a clear correlation between these parameters. A wide range of *T*_*2*_ values is present, but the range of diffusivities is more narrow. Apparently, the heterogeneity that is visible in the *T*_*2*_ maps does not translate to heterogeneity in the diffusivity. If the *T*_*2*_ values are mainly a function of local EPS content, the diffusivity is not correlated with EPS content. There are three possible explanations for the absence of a correlation: first, the diffusivity could be more impacted by the presence of microbial cells than by the presence of EPS. Second, the *T*_*2*_ values are influenced by the local amount of exchangeable protons (Hills, 1992). If the EPS properties are heterogeneous throughout the granule, the range of *T*_*2*_ values does not represent EPS content, but rather EPS properties. Third, the absence of a correlation can be due to the impact of paramagnetic impurities (e.g., metal ions, iron oxide, iron sulfides, and vivianite). It is known that these impurities have an impact on *T*_*2*_ (Brownstein & Tarr, 1979) and especially in the granule from full‐scale treatment plants inorganic contaminants might be present (Pronk et al., 2015). Their effect on *T*_*2*_ was, however, not assessed in this study. Previous researchers have reported a relation between *T*_*2*_ and diffusivity, although different NMR methods were used. Gonzalez‐Gil et al. (2001) found a cluster morphology in methanogenic granules. These clusters could be identified from stereomicroscopy images, *T*_*2*_ maps, and apparent diffusivity measurements. Similarly, Lens et al. (2003) observed a distribution of *T*_*2*_ values and diffusion coefficients in methanogenic granules. In neither of these publications, a (quantitative) correlation was reported. In line with the observations in the present study, Phoenix and Holmes (2008) reported relatively little diffusion heterogeneity for a structurally complex phototrophic biofilm. Furthermore, Herrling et al. (2017) found there was no clear relation between diffusion and *T*_*2*_ based on a D‐*T*_*2*_ experiment. Thus, *T*_*2*_ maps are of limited use for the characterization of diffusional properties, despite being relatively easy to obtain.

### 4.3 Implications for practice

Engineering of AGS reactors is, amongst others, based on conversion rates of different contaminants and on the flux of oxygen into the granules. Ideally, the flux of oxygen is sufficiently high to maintain an aerobic zone for nitrification, but also sufficiently low to maintain an anoxic core for denitrification (Mosquera‐Corral et al., 2005). The flux into a granule can be predicted with reaction‐diffusion models. In these models, there are two aspects of diffusion that should be considered: (a) using an accurate value for the diffusivity and (b) properly including diffusion heterogeneity. Regarding the first aspect, the question arises what accuracy is required. For most process engineering purposes, the flux into or out of a granule is the parameter of interest. According to half‐order kinetics (Harremoës, 1978), the overall substrate flux is proportional to the square root of the diffusion coefficient. This means that an error of 10% in the diffusion coefficient leads to an error in the flux of roughly 5%. Similar results have been found with the benchmark problem BM1 for different biofilm models (Morgenroth et al., 2004) and a local sensitivity analysis of IFAS and MBBR systems (Boltz et al., 2011). Thus, the uncertainty in the diffusion coefficient is not amplified. However, when the free water diffusion coefficient (∼2.0 × 10^−9^ m^2^/s) is used instead of the granule diffusion coefficient (∼1.4 × 10^−9^ m^2^/s) in an AGS model, an error is introduced of 20% in the flux over the granules surface. Incorrect conclusions may be drawn from the model, if this error is not accounted for (e.g., by fitting parameters as in Baeten, van Loosdrecht, & Volcke, 2018).

Regarding the second aspect, multiple authors have argued that heterogeneous diffusion should be incorporated into mathematical models of AGS (Chiu et al., 2007a; Liu et al., 2010; Tay et al., 2002). Their argument is generally based on the observation of heterogeneous granule structures with reduced or increased diffusivity, such as channels, layers, clusters, and pores. However, the results from the granule‐scale reaction‐diffusion model show that heterogeneous diffusion does not lead to a significantly different flux. This is most likely due to the fact that the average diffusion coefficient is maintained in our model. A higher diffusion coefficient in one part of the granule (e.g., channels) leads to a lower diffusion coefficient in the rest of the granule. Thus, the flux into a granule will only increase notably if the average diffusion coefficient over the whole granule increases. Most methods to study the diffusion behavior of a solute in granules, will yield an average diffusion coefficient (Chiu et al., 2007b; Fan, Leyva‐Ramos, Wisecarver, & Zehner, 1990; Horn & Morgenroth, 2006; Yu & Pinder, 1994). This average diffusion coefficient should always be maintained when constructing a biofilm model, to obtain a valid representation of the flux into a granule.

In contrast to our findings, other authors reported a large impact of a heterogeneous diffusion coefficient (Beyenal & Lewandowski, 2005; Morgenroth, Eberl, & van Loosdrecht, 2000; Siegrist & Gujer, 1985). The biofilms investigated by these authors were best described by a stratified diffusion coefficient. However, these biofilms were dense at the bottom and more porous toward the surface. The stratified diffusion coefficient was used to include the effect of advection (eddy diffusion) in the pores of the biofilm. Granules are more dense toward the surface and the surface is smooth (Chiu et al., 2007a; de Kreuk & Van Loosdrecht, 2004). Therefore, the effect of advection is expected to be negligible for granules.

## 5 CONCLUSION

In this study, diffusive mass transfer within lab‐scale and full‐scale AGS has been characterized with PFG‐NMR. The self‐diffusion coefficient of water inside the EPS matrix was roughly 70% of the diffusion coefficient in bulk water, for lab‐scale as well as for full‐scale AGSs. Despite the differences in operating conditions and influent characteristics, the differences in diffusion between lab‐scale and full‐scale granular sludges were only minor. The granules types differed in structure: while lab granules were more homogeneous, full‐scale granules were clearly heterogeneous. The latter consistently displayed irregular features such as voids and dense areas. However, no correlation between structural heterogeneity and diffusional properties was found. Despite the heterogeneous structure, the variation in diffusion coefficient for a single granule source was limited. A granule‐scale reaction‐diffusion model showed that small spatial variations in the diffusion coefficient do not lead to a large change (<1%) of substrate flux into the granule. Therefore, heterogeneity in diffusion does not play a major role in the conversion rates obtained with AGS.

Our study has several implications for modeling of the AGS process and for analysis of AGS kinetic properties. We recommend using a general diffusion coefficient that is 70% of the diffusion coefficient in water. Heterogeneity of diffusion on a granule‐scale does not need to be included to evaluate substrate flux into or out of a granule. Thus, a relatively simple approach is sufficient to describe mass transport by diffusion in AGS. Since we did not observe any difference between the different granular sludge types, this approach is most likely valid for all AGS plants that treat domestic wastewater.

## Supporting information

Supporting information
