# Supplementary material for: Heterogeneous diffusion in aerobic granular sludge
Source: Biotechnol Bioeng. 2020 Aug 6;117(12):3809–19. doi: 10.1002/bit.27522 (PMC7818175; doi:10.1002/bit.27522)
Supplement: Supplementary file 1 — Supporting information [file BIT-117-3809-s001.docx]

**Supplementary information**

**Heterogeneous Diffusion in Aerobic Granular Sludge**

Lenno van den Berg^1*^, Catherine, M. Kirkland^2,3^, Joseph D. Seymour^2,4^, Sarah L. Codd^2,5^, Mark C.M. van Loosdrecht^6^, Merle K. de Kreuk^1^

^1^ Department of Water Management, Delft University of Technology, The Netherlands

^2^ Center for Biofilm Engineering, Montana State University, Montana, USA

^3^ Department of Civil Engineering, Montana State University, Montana, USA

^4^ Department of Chemical and Biological Engineering, Montana State University, Montana, USA

^5^ Department of Mechanical and Industrial Engineering, Montana State University, Montana, USA

^6^ Department of Biotechnology, Delft University of Technology, The Netherlands

*Corresponding author. Stevinweg 1, 2628 CN Delft, The Netherlands. E-mail: [L.vandenBerg@tudelft.nl](mailto:L.vandenBerg@tudelft.nl)

**Stejskal-Tanner plot of a typical diffusion experiment**

| **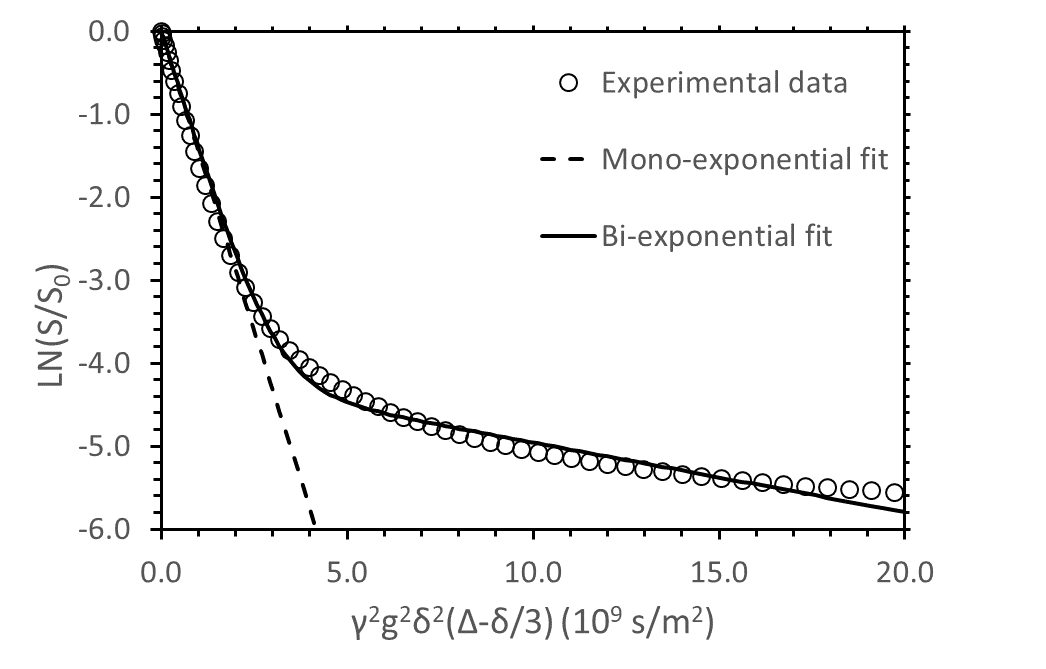** |
| --- |
| **Figure S1. Stejskal-Tanner plot of the normalized NMR signal as function of the b-value, where b is defind as** $\boldsymbol{\gamma}^{\boldsymbol{2}}\boldsymbol{g}^{\boldsymbol{2}}\boldsymbol{\delta}^{\boldsymbol{2}}\left( \boldsymbol{\Delta-}\frac{\boldsymbol{\delta}}{\mathbf{3}} \right)$**. The bi-exponential behaviour of the experimental data is apparent. The experimental data in this plot is from a Garmerwolde granule sample.** |

**Probability distributions of the diffusion coefficient**

| **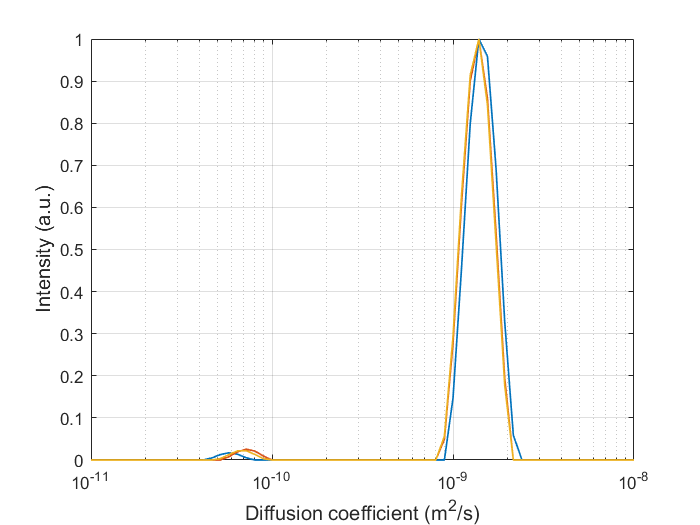** |
| --- |
| **Figure S2. Diffusion coefficient pobability distribution of three Nereda Garmerwolde samples. The small differences between the triplicate samples is clear. Also note the presence of a smaller peak around 5 · 10^-11^ m^2^/s.** |
| **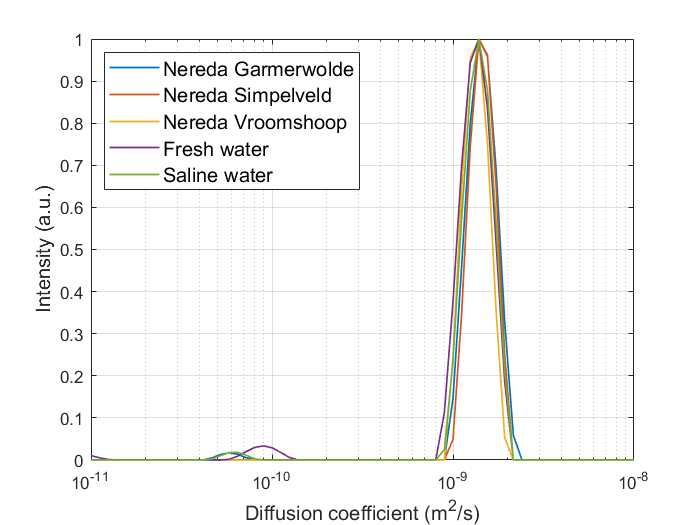** |
| **Figure S3. Diffusion coefficient pobability distribution of five different granule samples. The samples are highly comparable on the scale of this figure.** |

**Effective *T_2_* maps and D-*T_2_* correlations for five different granule types**

For all the granular sludge sources investigated, effective *T_2_* maps and D-*T_2_* correlations were made. These maps and correlations reveal information on the granule structure and possible relationship with diffusion in the granule (see the main paper for a more detailed explanation). Per granule source, multiple granules were imaged to obtain multiple *T_2_* maps. Here, only a single *T_2_* map is reported, which is considered typical of the granule source. For the *T_2_* maps lighter regions correspond to a higher *T_2_* (more water-like), while darker regions correspond to a lower *T_2_* (more solid-like). The D-*T_2_* correlations are made for a granular sludge sample (test tube packed with >> 10 granules) and only a single D-*T_2_* correlation was made.

| 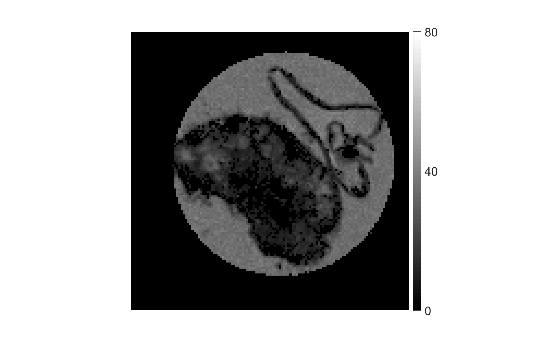  4.1 mm | 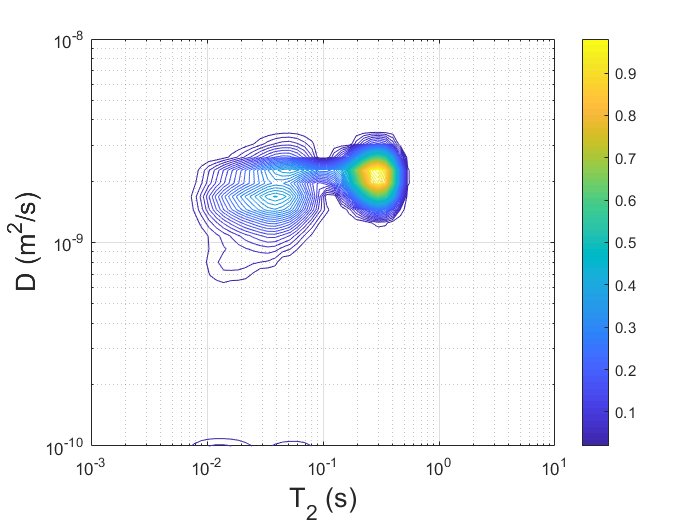 |
| --- | --- |
| **Figure S3. Typical effective *T_2_* map for a Garmerwolde full-scale granule (left), with the scale bar indicating effective *T_2_* in ms. The spatial resolution is 39 x 39 x 100 µm. Typical D-*T_2_* correlation for Garmerwolde full-scale granular sludge (right). The colour bar represents signal intensity in arbitrary units.** | |
| 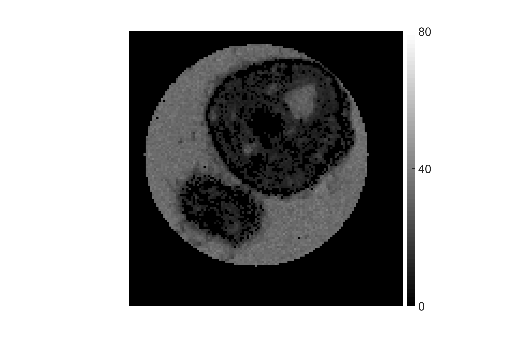  4.1 mm | 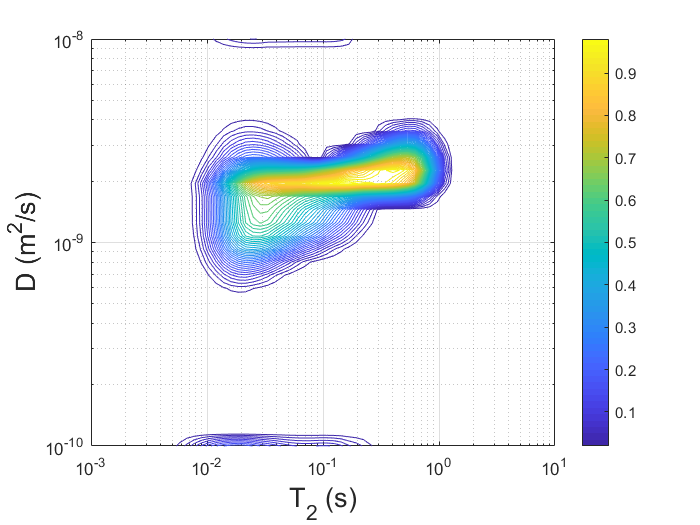 |
| **Figure S4. Typical effective *T_2_* map for a Simpelveld full-scale granule (left), with the scale bar indicating effective *T_2_* in ms. The spatial resolution is 39 x 39 x 100 µm. Typical D-*T_2_* correlation for Simpelveld full-scale granular sludge (right). The colour bar represents signal intensity in arbitrary units.** | |
| 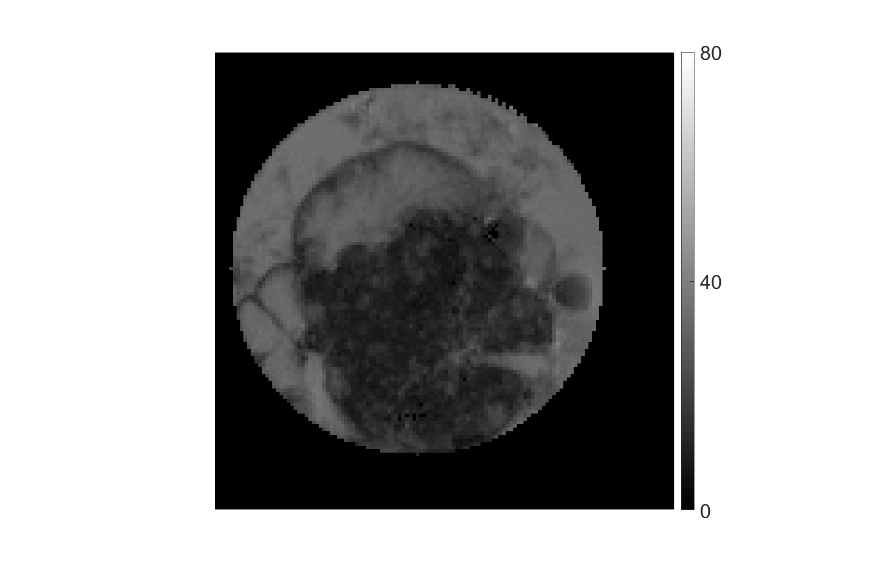  4.1 mm | 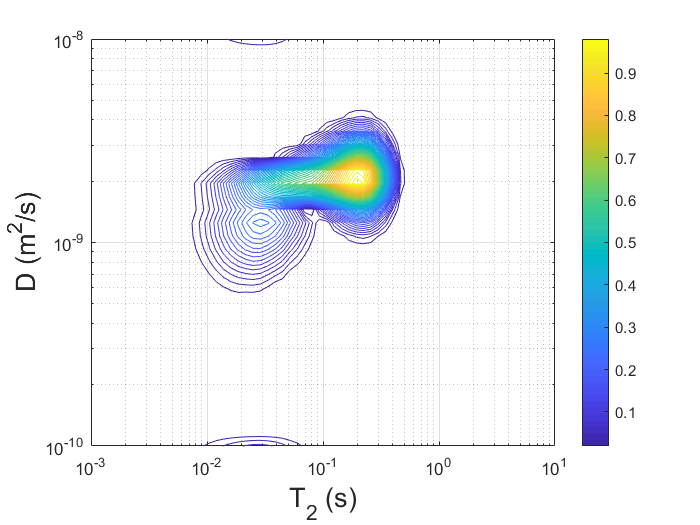 |
| **Figure S5. Typical effective *T_2_* map for a Vroomshoop full-scale granule (left), with the scale bar indicating effective *T_2_* in ms. The spatial resolution is 39 x 39 x 100 µm. Typical D-*T_2_* correlation for Vroomshoop full-scale granular sludge (right). The colour bar represents signal intensity in arbitrary units.** | |
| 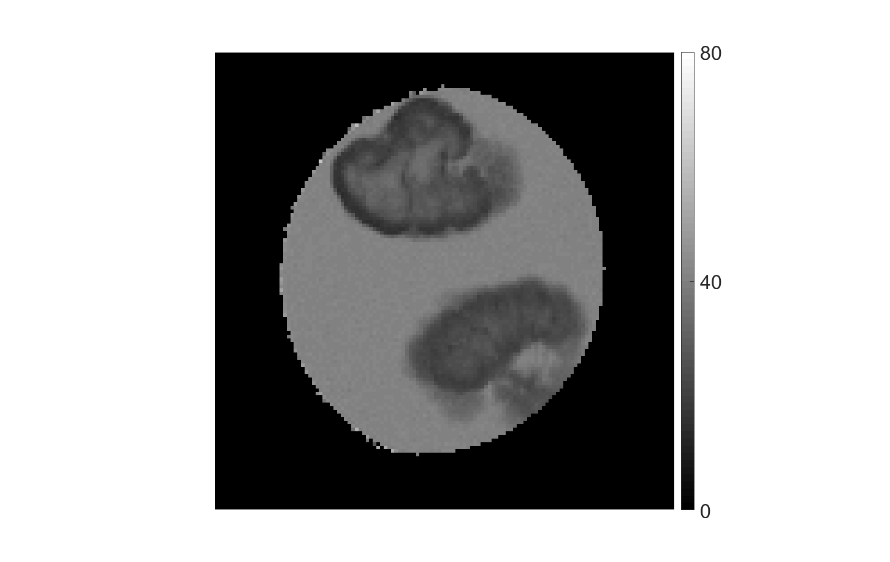  4.1 mm | 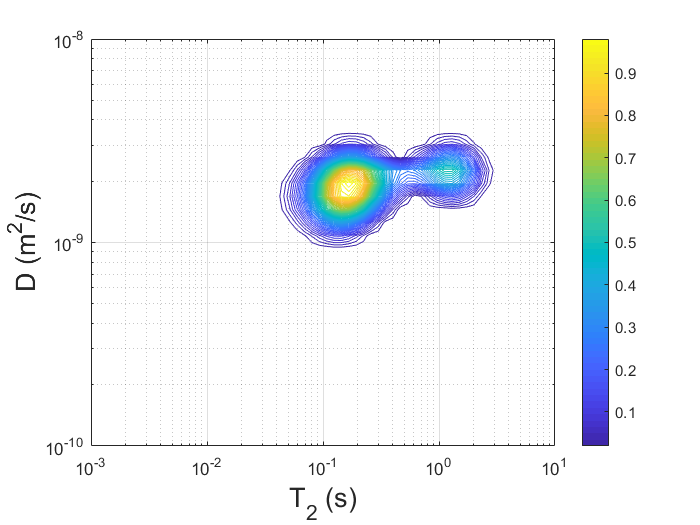 |
| **Figure S6. Typical effective *T_2_* map for a freshwater lab-scale granule (left), with the scale bar indicating effective *T_2_* in ms. The spatial resolution is 39 x 39 x 100 µm. Typical D-*T_2_* correlation for freshwater lab-scale granular sludge (right). The colour bar represents signal intensity in arbitrary units.** | |
| 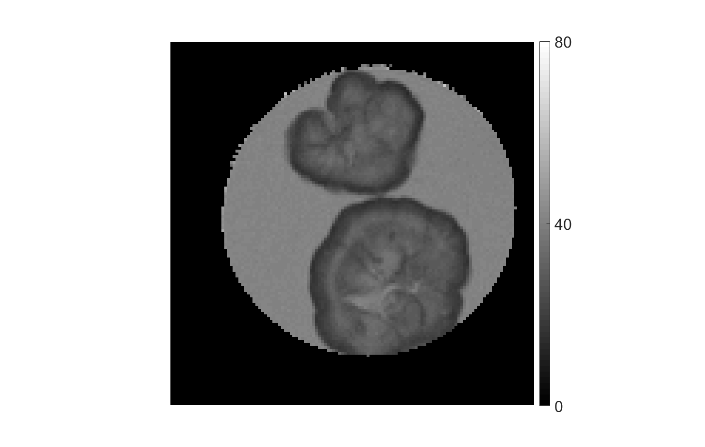  4.1 mm | 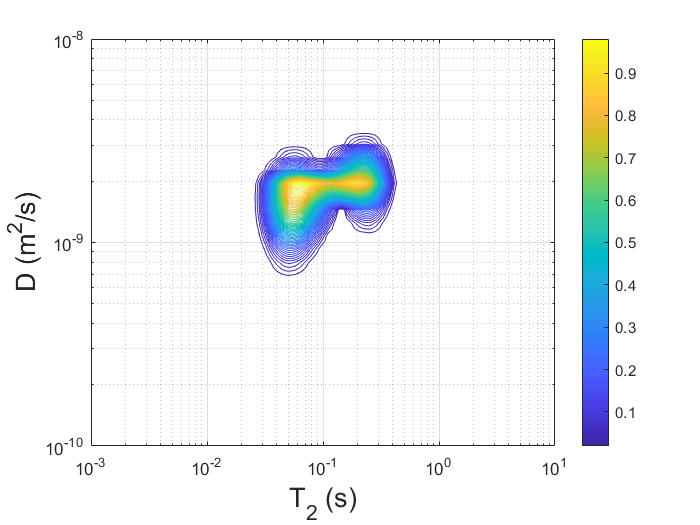 |
| **Figure S7. Typical effective *T_2_* map for a saline lab-scale granule (left), with the scale bar indicating effective *T_2_* in ms. The spatial resolution is 39 x 39 x 100 µm. Typical D-*T_2_* correlation for saline lab-scale granular sludge (right). The colour bar represents signal intensity in arbitrary units.** | |

**Relation between effective diffusion coefficient and simulated flux**

| **** |
| --- |
| **Figure S8. Relation between the diffusion coefficient of the penetrated volume (labeled here as effective diffusion coefficient) and the flux deviation (the difference in flux between a heterogeneous diffusion scenario and homogeneous diffusion). The data points (solid circles) correspond to all six heterogeneity scenarios, with bulk oxygen concentrations of 0.5, 1.0, 1.5, 2.0, 3.0, 5.0, 7.5, and 10.0 mg/L. The good correlation is apparent from the R^2^ of the linear trend line (solid line). The dashed lines correspond to the homogeneous diffusion case.** |
